# Synthesis of Mesoporous Silica Adsorbent Modified with Mercapto–Amine Groups for Selective Adsorption of Cu^2+^ Ion from Aqueous Solution

**DOI:** 10.3390/nano12183232

**Published:** 2022-09-18

**Authors:** Sagar M. Mane, Chaitany Jayprakash Raorane, Jae Cheol Shin

**Affiliations:** 1 Division of Electronics and Electrical Engineering, Dongguk University-Seoul, Seoul 04620, Korea; 2 School of Chemical Engineering, Yeungnam University, Gyeongsan 38541, Korea

**Keywords:** silica nanoparticles, click chemistry, adsorption, aqueous solution, desorption

## Abstract

In a sol–gel co-condensation, a mesoporous silica hybrid integrated with (3-mercaptopropyl)trimethoxysilane (TMPSH) was prepared and then reacted with allylamine via a post-surface functionalization approach. Approximately 15 mol% of TMSPSH was introduced into the mesoporous silica pore walls along with tetraethyl orthosilicate. The mercapto ligands in the prepared mesoporous silica pore walls were then reacted with allylamine (AM) to form the mercapto–amine-modified mesoporous silica adsorbent (MSH@MA). The MSH@MA NPs demonstrate highly selective adsorption of copper (Cu^2+^) ions (~190 mg/g) with a fast equilibrium adsorption time (30 min). The prepared adsorbent shows at least a five times more efficient recyclable stability. The MSH@MA NPs adsorbent is useful for selective adsorption of Cu^2+^ ions.

## 1. Introduction

With the rapid development of the industrial sector, heavy metal pollutants are now considered a serious concern [1,2]. Toxic metal ions including copper, lead, mercury, etc., contaminate water bodies, and they induce severe biological and environmental toxicity [3,4]. With high solubility and bioaccumulation, copper ions (Cu^2+^) are considered toxic pollutants above a certain level. Copper poisoning can harm the immune system of living things, and cause disabilities to internal organs [5,6,7,8]. Among the various techniques available, adsorption is the most widely applied technique used for Cu^2+^ ion separation [9,10,11,12,13]. Designing novel adsorbent materials desirable for toxic metal ions adsorption is needed urgently. In general, porous materials are considered economically feasible. A range of materials including mesoporous carbon and silica materials are used for toxic metal ions adsorption [14,15,16,17,18,19]. Amongst these, mesoporous silica has proven significant adsorption efficiency due to its porous structure, high surface area, and recyclability [20,21,22,23,24]. Further, mesoporous silica materials offer the space to incorporate functional ligands to enhance adsorption capacity [25,26,27].

Mesoporous silica functionalized with mercapto groups (-SH) and amine groups (-NH_2_) show a specific affinity for specific metal ions [28,29,30,31]. In general, increasing the ligand group content in adsorbent materials is thought to significantly improve the adsorption efficiency [32,33,34]. Sol–gel is the most simple, cost-effective, and very useful method to develop these kinds of materials. The co-condensation synthetic method offers the advantage of being able to introduce a high amount of organic functional ligand content in the mesopore walls [35,36,37].

In this work, we synthesize the mercapto–amine-groups-modified mesoporous silica adsorbent via a co-condensation method, and surface modification to increase the application efficiency of the adsorbent towards selective metal ions (Cu^2+^ ions). The synthesized mercapto–amine-grafted mesoporous silica adsorbent (MSH@MA NPs) was analyzed and used to evaluate the removal capacity of Cu^2+^ ions from water. In addition, the adsorption characteristics of the prepared MSH@MA NPs adsorbent were evaluated by studying various parameters including pH, time, metal ion concentration, and recyclability. The study results suggest that the MSH@MA NPs could be used to selectively remove Cu^2+^ ions from contaminated water.

## 2. Materials and Methods

### 2.1. Chemicals

Pluronic P123 (average Mn 5800), (3-mercaptopropyl)trimethoxysilane (TMSPSH, 95%), tetraethoxysilane (TEOS, 98%), allylamine (AM, 98%), Cu(NO_3_)_2_·3H_2_O (98%), Cd(NO_3_)_2_·4H_2_O (98%), Pb(NO_3_)_2_ (99%), Cr(NO_3_)·6H_2_O, tetrahydrofuran (THF), hydrochloric acid (HCl), ethanol, and deionized water were received from Aldrich Chemicals, St. Louis, MO, USA. All the metal ion stock solution was prepared with 100 mg/mL by dissolving the appropriate amount of each metal salt in a neutral pH (pH 7) buffer and stored at 4 °C.

### 2.2. Synthesis of MSH@SH NPs

**Scheme 1:**
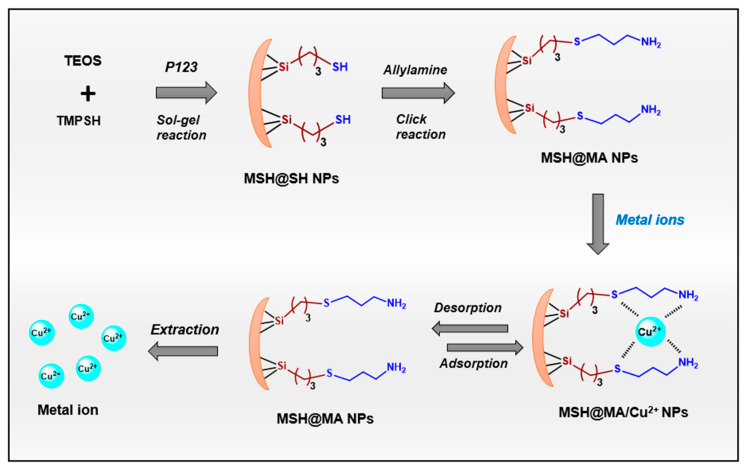
Schematic representation for the synthesis of MSH@MA NPs adsorbent.

For the synthesis of MSH@SH NPs, first, the (3-mercaptopropyl)trimethoxysilane (TMSPSH)-groups-incorporated mesoporous silica was created via co-condensation method using Pluronic P123 as structure-directing templating agent. To form a clear solution, P123 (1 g) was dissolved in deionized water (40 mL) containing 3 g HCl under magnetic agitation for 2 h at 37 °C. The pre-mixed TMPSH and TEOS solution [TMPSH/(TEOS + TMPSH) = 15 mol%] was slowly added to this, and the solution was agitated for 24 h at 40 °C, and further heated at 90 °C. The obtained precipitate was filtered, washed with water, and dried at 60 °C. The surfactant was extracted by stirring with an acidified ethanolic solution (1 mL HCl in 50 EtOH). The extraction procedure was carried out three times. MSH@SH NPs was the name given to the extracted surfactant sample (Scheme 1). Second, the mercapto groups in the MSH@SH sample were further modified with allylamine (AM) groups (5 mL, 0.2 M) by incubating the MSH@SH NPs (0.2 g) in ethanol (20 mL) for 24 h at 55 °C [38]. The finished product was filtered, washed with THF and ethanol, and dried at 60 °C. MSH@MA NPs was the name given to the mercapto–amine-modified mesoporous silica adsorbent (Scheme 1). For comparison, we prepared control MSNs without any ligand functionalities such as mercapto–amine (MA) groups. For the synthesis of a control sample, about 1 g of P123 was dissolved in deionized water (40 mL) containing 3 g HCl under magnetic agitation for 2 h at 37 °C. To this, TEOS solution (1.5 mL) was added, and the solution was magnetically stirred for 24 h at 40 °C and further heated at 90 °C. The obtained precipitate was filtered, washed with water, and dried at 60 °C. The surfactant was extracted by stirring with an acidified ethanolic solution (1 mL HCl in 50 EtOH). The obtained sample was named as MSNs.

### 2.3. Characterization

Fourier-transform infrared (FT-IR, JASCO FTIR 4100, Easton, MD, USA) spectra were carried out using the KBr pelleting method. X-ray diffraction (XRD, Bruker AXN, Billercia, MA, USA) analysis was performed with CuK radiation. Nitrogen adsorption–desorption experiments were carried out on a Nova 4000e analyzer (Quantachrome Instruments, Boynton Beach, FL, USA) at −196 °C. The surface area, pore size, and pore volumes were calculated using the Brunauer–Emmett–Teller (BET) and Barrett–Joyner–Halenda (BJH) techniques, respectively. Scanning electron microscopy (SEM, JEOL 6400, Akishima, Tokyo, Japan) was performed with a 10 kV accelerating voltage. A transmission electron microscope (TEM) analysis was performed on a JEOL 2010 TEM, Akishima, Tokyo, Japan at 200 kV. The materials were subjected to thermogravimetric (TG, Perkin-Elmer Pyris 1, Waltham, MA, USA) analysis at a heating rate of 10 °C/min. An inductively coupled plasma atomic emission spectrometer was used to quantify adsorbed metal ions (ICP-AES, Perkin-Elmer, Model Optima 5300 DV, Waltham, MA, USA).

### 2.4. Adsorption Study

The following equations were applied to determine the amounts of an adsorbed metal ion at equilibrium (Qe, mg/g) and at time t (Qt, mg/g).

Q_e_ = [(C_0_ − C_e_)V]/m (1)

Qt = [(C_0_ − Ct)] V/m (2)

where C_0_ and C_e_ are the initial and equilibrium concentrations, respectively, and C_t_ is the concentration at any given time t; V is the volume of solution (mL), and m is the adsorbent weight (g).

#### 2.4.1. Effect of pH

To carry out this study, MSH@MA NPs sample (0.1 g) was suspended in a 10 mL buffer solution and about 0.5 mL of Cu^2+^ ion solution (100 mg/L) was introduced. Further, the solution medium was adjusted with different pH levels (3, 5, 6.5, 7, and 9) using 0.1 M HCl, and shaken at 25 °C for 60 min to reach equilibrium. The sample was isolated by filtration, and the obtained filtrate solution was used to determine unadsorbed Cu^2+^ ions using the ICP-AES instrument.

#### 2.4.2. Effect of Time

A kinetic adsorption study was performed at different time points. For this study, the MSH@MA NPs sample (0.1 g) was suspended in water (10 mL), and the medium pH was adjusted. Cu^2+^ ion solution (0.5 mL) was introduced and agitated at 25 °C for different lengths of time (5–120 min). After the specified time, the sample was filtered and washed with water. ICP-AES analysis was performed on the filtrate obtained.

#### 2.4.3. Effect of Competitive Metal Ions

For this study, the MSH@MA NPs sample (0.1 g) was suspended in a vial containing 10 mL of a mixed solution containing Cu^2+^ and Co^2+^, Cd^2+^, Pb^2+^, and Cr^3+^ ions, and then agitated at 25 °C for 60 min. Finally, ICP-AES analysis was used to determine the number of metal ions adsorbed.

#### 2.4.4. Recyclability Test

To accomplish this study, metal-adsorbed MSH@MA/Cu^2+^ NPs sample (0.1 g) was agitated for 60 min at 25 °C in 10 mL of aqueous HCl solution (0.1 M). The sample was filtered, washed several times, and dried at 60 °C. The desorbed metal ion was determined. The adsorption–desorption procedure was repeated five times in a row, with the adsorption efficiency of the MSH@MA NPs being determined each time.

## 3. Results and Discussion

### 3.1. FTIR Analysis

**Figure 1:**
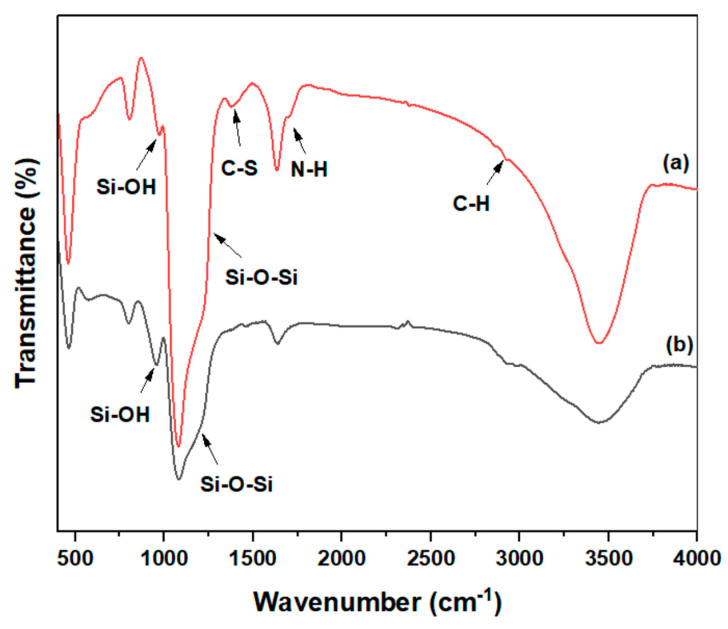
FTIR spectra of the (**a**) MSH@MA NPs; (**b**) MSNs.

The FTIR spectrum of mercapto-amine-groups-functionalized mesoporous silica MSH@MA NPs is shown in Figure 1a. The stretching modes at 594, 2622, 2895, and 2932 cm^−1^, are assigned to the stretching modes of the alkyl (-CH_2_-CH_2_-) stretching peaks, evidencing that the mercapto-amine groups are present in the silica materials [39,40]. In addition, the typical band at 965 and 1088 cm^−1^ promotes the formation of surface silanol and siloxane networks in MSH@MA NPs. Moreover, the control MSNs show at 965 cm^−1^ and 1088 cm^−1^, depicting surface silanol and siloxane groups, without any specific peaks for organic ligand mercapto-amine groups (Figure 1b).

### 3.2. XRD and BET Analysis of MSH@MA NPs

**Figure 2:**
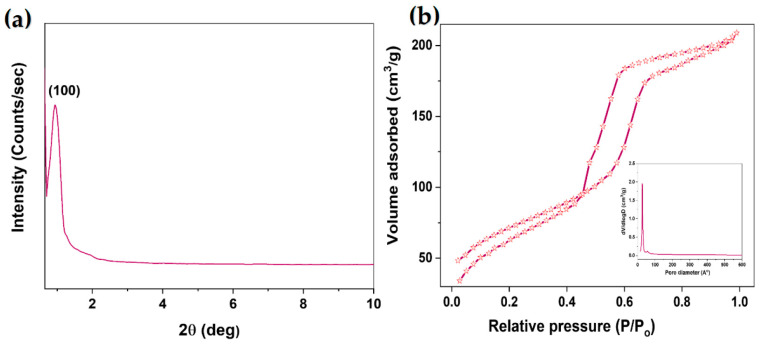
Structural and surface area analysis. (**a**) Low-angle XRD pattern of the MSH@MA NPs. (**b**) N_2_ adsorption–desorption isotherm and pore size distribution (inset figure) of the MSH@MA NPs.

Figure 2a depicts the powder XRD pattern of the mercapto–amine-groups-functionalized MSH@MA NPs. The sample shows the diffraction peak at 2θ = 0.97°, indicating (100) diffraction that indicates the formation of an ordered mesostructure arrangement in the MSH@MA NPs, as shown in Figure 2a. A similar XRD pattern is also observed for the control samples (See Figure S1 in Supplementary Materials). Figure 2b shows type IV isotherm curves with H1 hysteresis of the MSH@MA NPs sample, indicating mesopore formation [41]. The MSH@MA NPs sample’s surface area, pore volume, and mesopore size are determined to be approximately 563 m^2^/g, 0.61 cm^3^/g, and 2.7 nm, respectively. For the control MSNs, the estimated surface area, pore volume, and average mesopore size are determined to be 690 m^2^/g, 0.72 cm^3^/g, and 3.1 nm, respectively (See Figure S2a in Supplementary Materials).

### 3.3. TGA, Zeta, and Particle Size Analysis of MSH@MA NPs

**Figure 3:**
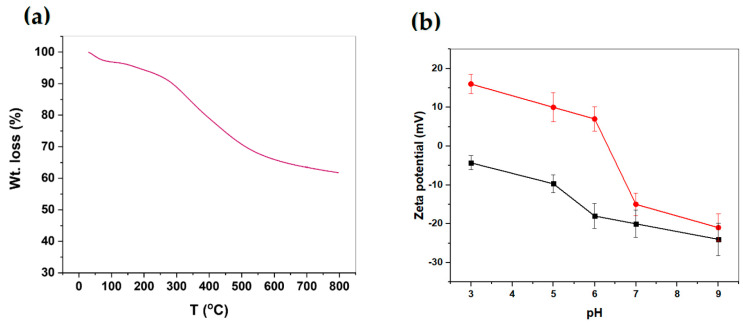

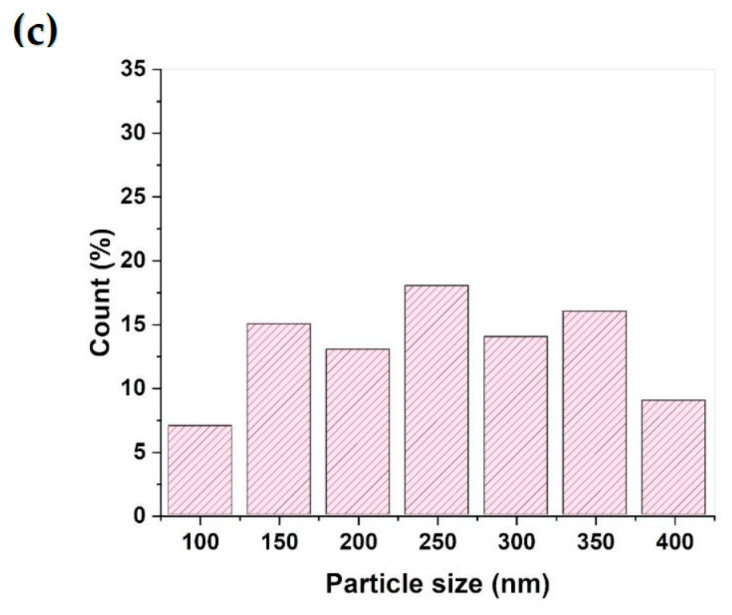
(**a**) TG analysis of MSH@MA NPs. (**b**) Zeta potential of MSH@MA NPs (red line) and MSNs (black line). (**c**) Particle size distribution of the MSH@MA NPs.

The TG analysis of the MSH@MA NPs is shown in Figure 3a. As shown in Figure 3a, the MSH@MA NPs sample experiences an initial weight loss of about 2.3 wt% at 100 °C, due to surface-adsorbed moisture evaporation. Following that, a 20.8 wt% weight loss occurs at 101–650 °C, indicating that the functionalized ligand decomposes. The control MSNs sample shows a negligible weight loss, with an overall weight loss of about ~2.8 wt% caused by physisorbed moisture (See Figure S3a). As shown in Figure 3b, the MSH@MA NPs sample has positive zeta potential values of about +16 ± 1 mV, +10 ± 2 mV, and +7 ± 1 mV at pH 3, 5, and 6 respectively. In addition, the zeta potential values at pH 7 and 9 are about −15 ± 1 and −21 ± 2 mV, respectively, and the negative zeta potential value arises due to the presence of sulfur atom in the modified functional groups, which supports the existing mercapto–amine ligands in the MSH@MA NPs adsorbents [42]. The control sample shows negative zeta potential values of about −6 to −24 mV at pH 3 to 9, respectively, indicating the presence of negatively charged silanol groups (Figure 3b). Furthermore, the particle size analysis of MSH@MA NPs measured by DLS analysis reveals particle sizes fall in between the 100 to 400 nm range, with the average particle size of about ~250 ± 100 nm (Figure 3c). In contrast, MSNPs show particle sizes in the range from ~100 nm to 500 nm, with an average particle size of about ~300 nm (See Figure S3b).

### 3.4. Morphological Analysis (SEM and TEM) of MSH@MA NPs

**Figure 4:**
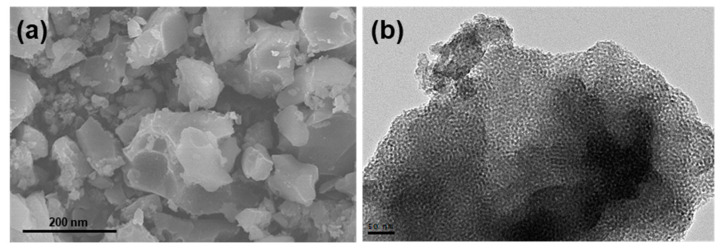
Morphological analysis (**a**) SEM; and (**b**) TEM images of the MSH@MA NPs.

SEM images of MSH@MA NPs reveal aggregated particles with an average size of 400 nm, as shown in Figure 4a. The TEM image (Figure 4b) shows the mesopore arrangement in the MSH@MA NPs sample, evidencing the existence of ordered mesopore structures. The presence of wormhole-like porous structure can be easily noticed from the TEM analysis. SEM and TEM (inset) images of the MSNs sample also show aggregated particles with a particle size of about 350 nm, with clear mesopore channels (See Figure S2b, ESI).

### 3.5. Adsorption Kinetics

#### 3.5.1. Effect of pH and Time on Adsorption Efficacy of MSH@MA NPs

**Figure 5:**
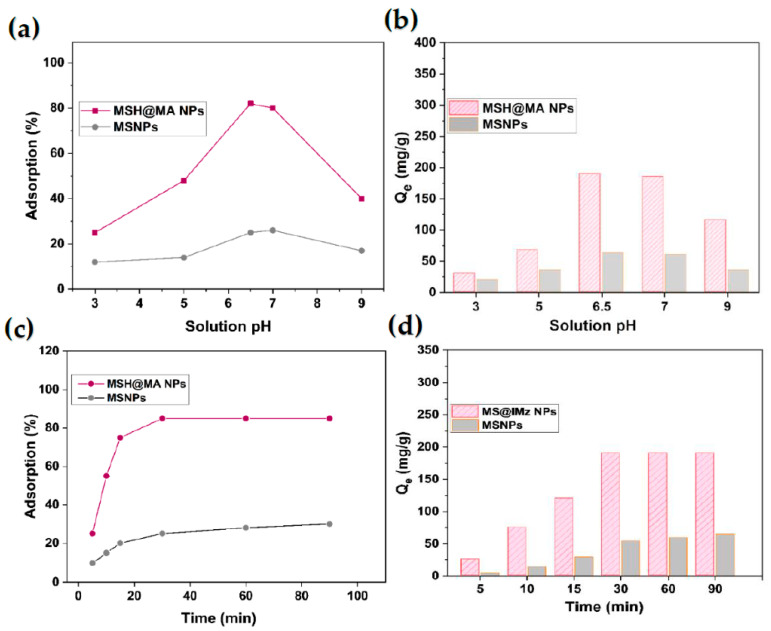
Adsorption kinetics of MSH@MA NPs. (**a**) Influence of solution pH on the adsorption percentage of Cu^2+^ ions onto the MSH@MA NPs. (**b**) Influence of solution pH on the amount of adsorption of Cu^2+^ ions onto the MSH@MA NPs. (**c**) Influence of contact time on the adsorption percentage of Cu^2+^ ions onto the MSH@MA NPs. (**d**) Influence of contact time on the amount of adsorption of Cu^2+^ ions onto the MSH@MA NPs.

Adsorption medium pH-based adsorption was studied at different pH conditions. Figure 5a shows that the Cu^2+^ ion adsorption gradually increases from pH 3 to pH 7, and then decreases further while reaching pH 9. The maximum of Cu^2+^ ions adsorption is achieved at pH 6.5, the estimated amount being 190 mg/g (Figure 5b). Therefore, pH 6.5 was selected for further kinetic studies. Furthermore, the MSH@MA NPs show enhanced adsorption in the pH 3 to 7 range, indicating that the surface-modified mercapto–amine groups effectively increase the adsorption efficiency of the MSH@MA NPs. The adsorption capacity of Cu^2+^ ions decreases at a low pH due to lower metal ion–ligand affinity. This might be caused due to the interference of H^+^ ions and the Cu^2+^ ions under reduced pH [43]. The adsorption efficiency of MSH@MA NPs towards Cu^2+^ ions is greatly decreased at low pH conditions such as pH 3 and pH 5. At these reduced pH conditions, the concentration of acidic protons (H^+^ ions) in the solution medium is considerably higher and it could competitively interact with the nitrogen (N) and sulfur (S) atoms. Thus, the Cu^2+^ ion interaction with these mercapto–amine ligand groups is considerably hindered. This is the reason why the Cu^2+^ ions are adsorbed at a low amount by the MSA@MA NPs under acidic pH conditions [44].

Time-dependent adsorption kinetics were calculated using a Cu^2+^ ion solution containing 100 mg/L. Figure 5c shows that the adsorption efficiency of MSH@MA NPs on Cu^2+^ ions increases before reaching equilibrium. The prepared MSH@MA NPs takes a short time to reach maximum adsorption of 190 mg/g, with approximately 85% of adsorption occurring within 30 min, and reaching equilibrium adsorption in about 60 min, and almost reaching equilibrium when increasing the time to 90 min (Figure 5d).

#### 3.5.2. Effect of Adsorbent Dosage and Selective Efficiency of MSH@MA NPs

**Figure 6:**
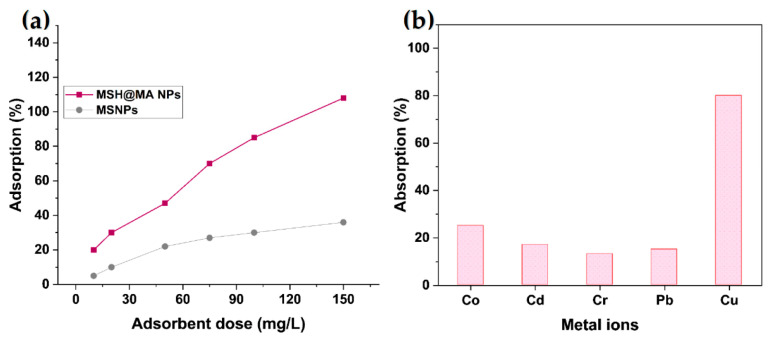
(**a**) Effect of adsorbent dosage of Cu^2+^ ions onto the MSH@MA and MS NPs. (**b**) Selective adsorption efficiency for various competitive metal ions by MSH@MA NPs.

The adsorption behavior of the prepared MSH@MA NPs was tested at 25 °C with different amounts of adsorbent (10–150 mg/L) using Cu^2+^ ions (100 mg/L). Figure 6a shows that increasing the quantity of MSH@MA NPs increases the Cu^2+^ ion adsorption. As shown in the graph, using adsorbent amounts of 100 mg/L, approximately 85% of the removed Cu^2+^ ions are adsorbed. The obtained results reveal that the quantity of mercapto–amine groups increases with increasing the adsorbent dosage of 150 mg/L, and the functionalities are responsible for the Cu^2+^ ion removal from the aqueous solution.

The selectivity of the adsorbent was studied along with the competitive metal ions. As shown in Figure 6b, the MSH@MA NPs sample exhibits more Cu^2+^ ion adsorption than the other metal ions. According to these findings, other competitive metal ions do not interfere with the modified functional groups on the MSH@MA NPs, and do not cause any notable adsorption efficiency toward the selective adsorption of Cu^2+^ ions. This implies that the mercapto–amine functional groups in MSH@MA NPs have a preferable affinity for Cu^2+^ ions than the other ions [23,44]. This is due to the interaction between Cu^2+^ ions and mercapto–amine being stronger than those between other metal ions and mercapto–amine ligands. Therefore, the higher selectivity of MSH@MA NPs towards Cu^2+^ ions is due to the fact that the softer transition metal ions (for example, Cu^2+^) are prone to form stable complexes with ligands carrying softer donor atoms [45].

#### 3.5.3. Adsorption Kinetics in Aqueous Solution and Recyclability of MSH@MA NPs

**Figure 7:**
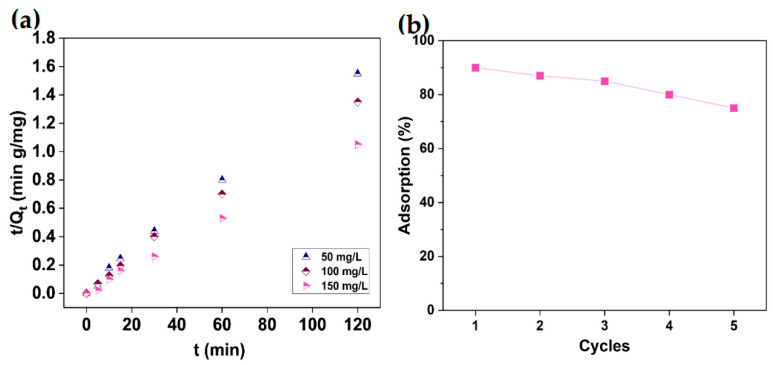
(**a**). Adsorption kinetics of the MSH@MA NPs in an aqueous solution of Cu^2+^ ions at an initial concentration of 50, 100, and 150 mg/L, respectively. (**b**) Adsorption–desorption efficiency towards Cu^2+^ ions in different cycles by MSH@MA NPs.

The adsorption of Cu^2+^ ions fit well with a pseudo-second-order kinetic equation in Figure 7a, and the constant Kad is estimated to be 3.9 × 10^−3^ g/mg/min and 4.8 × 10^−3^ g/mg/min for initial concentrations of 50 and 100 mg/L of Cu^2+^ ions, respectively (Table 1). The correlation coefficients (R^2^) for the Langmuir model are larger and fit better than the Freundlich model, indicating that the Cu^2+^ ions adsorption of the functional groups could be described as monolayer adsorption (Table 2). According to Table 3, the maximum adsorption capacity of MSH@MA NPs for Cu^2+^ is determined to be 190 mg/g, which is higher than the various types of adsorbents previously reported [43,44,45,46,47,48,49,50].

The recyclability and reusability of the prepared MSH@MA NPs were determined. For this study, 0.1 g of Cu^2+^ ions adsorbed MSH@MA NPs was treated with 0.1 M HCl (25 mL) and stirred for 60 min before centrifuging, washing with water, and drying. The adsorption capacity of the recycled sample was then tested by adsorbing the Cu^2+^ ions from the solution and determining the adsorbed metal ions. Figure 7b depicts the adsorption capacity maintained at about 95–80% in the subsequent three cycles, revealing the MSH@MA NPs’ retention of Cu^2+^ ions adsorption capacity.

## 4. Conclusions

In this study, mercapto–amine-groups-modified MSH@MA NPs were synthesized via thiolene click reaction for selective removal of Cu^2+^ ions. By this approach, enriched mercapto–amine functional groups were incorporated into the silica surfaces, which show selectivity towards Cu^2+^ ions. The experimental results reveal that solution pH, adsorption time, metal ion concentration, and adsorbent dosage all have a significant impact on adsorption behavior. At 25 °C, the prepared MSH@MA NPs have an estimated adsorption capacity of 190 mg/g of Cu^2+^ ions. The experimental adsorption results show that functionalized mercapto–amine groups in MSH@MA NPs have significantly higher adsorption activity towards Cu^2+^ ions. As a result, the MSH@MA NPs could be used to selectively remove Cu^2+^ ions from an aqueous solution.

## Figures and Tables

**Table 1:** The pseudo-second-order rate constant of adsorption kinetics of MSH@MA NPs in an aqueous solution of Cu^2+^ ions.

Initial Conc. of Metal Ions (mg/L)	K_ad_ (g/mg/min)	R^2^
50	Cu^2+^	Cu^2+^
	2.8 × 10^−3^	0.98
100	3.9 × 10^−3^	0.97
150	4.8 × 10^−3^	0.97

**Table 2:** Comparison of Langmuir and Freundlich isothermal adsorption-related parameters of the MSH@MA NPs materials for Cu^2+^ ions.

Langmuir				Freundlich		
	Q_m_ (mg/g)	K_L_ (L/mg)	R^2^	1/n	K_F_ ((mg/g)/(mg/mL))	R^2^
MSH@MA NPs/Cu^2+^	190	7.85	0.98	1.73	4.8	0.95

**Table 3:** Comparison of the adsorption capacity of the various functional adsorbent materials.

Type of Adsorbent	Adsorption_max_ (mg/g)	Reference
Sulfur-functionalized silica microspheres	62.3	[43]
Amino-functionalized mesoporous silica	36.4	[44]
Mesoporous silica SBA-15-triethylenetetramine	23.9	[45]
Multi-modified granulated SBA-15	31.8	[46]
Dopamine-functionalized silica NPs	58.7	[47]
Fe_3_O_4_@SiO_2_ nanoparticles	51.04	[48]
Chitosan–carboxymethyl starch composite	95	[49]
Chitosan@TiO_2_ composites	31	[50]
Mercapto–amine-modified mesoporous silica adsorbent	190	This work

## Data Availability

Not applicable.

## Supplementary Materials

The following supporting information can be downloaded at: https://www.mdpi.com/article/10.3390/nano12183232/s1, Figure S1: XRD analysis of control MSNs sample; Figure S2: (a) N_2_ sorption analysis; and (b) SEM and TEM (inset) images of control MSNs sample; Figure S3: (a) TGA; and (b) DLS analysis of control MSNs sample.
