# Supplementary material for: Synthesis of Mesoporous Silica Adsorbent Modified with Mercapto–Amine Groups for Selective Adsorption of Cu2+ Ion from Aqueous Solution
Source: Nanomaterials (Basel). 2022 Sep 18;12(18):3232. doi: 10.3390/nano12183232 (PMC9503849; doi:10.3390/nano12183232)
Supplement: Supplementary file 1 [file nanomaterials-12-03232-s001.zip › nanomaterials-1920002-supplementary.pdf]

nanomaterials

MDPI

Supplementary Materials

Synthesis of Mesoporous Silica Adsorbent Modified with Mercapto–Amine Groups for Selective Adsorption of Cu^{2+} Ion from Aqueous Solution

Sagar M. Mane [1], Chaitany Jayprakash Raorane [2,*] and Jae Cheol Shin [1,*]

[1] Division of Electronics and Electrical Engineering, Dongguk University-Seoul, Seoul 04620, Korea
[2] School of Chemical Engineering, Yeungnam University, Gyeongsan 38541, Korea
* Correspondence: chaitanyaraorane22@gmail.com (C.J.R.); jcshin@dgu.ac.kr (J.C.S.)

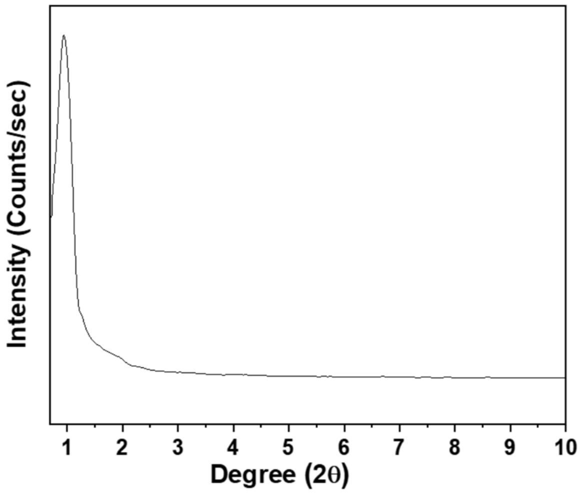

Figure S1. XRD analysis of control MSNs sample.

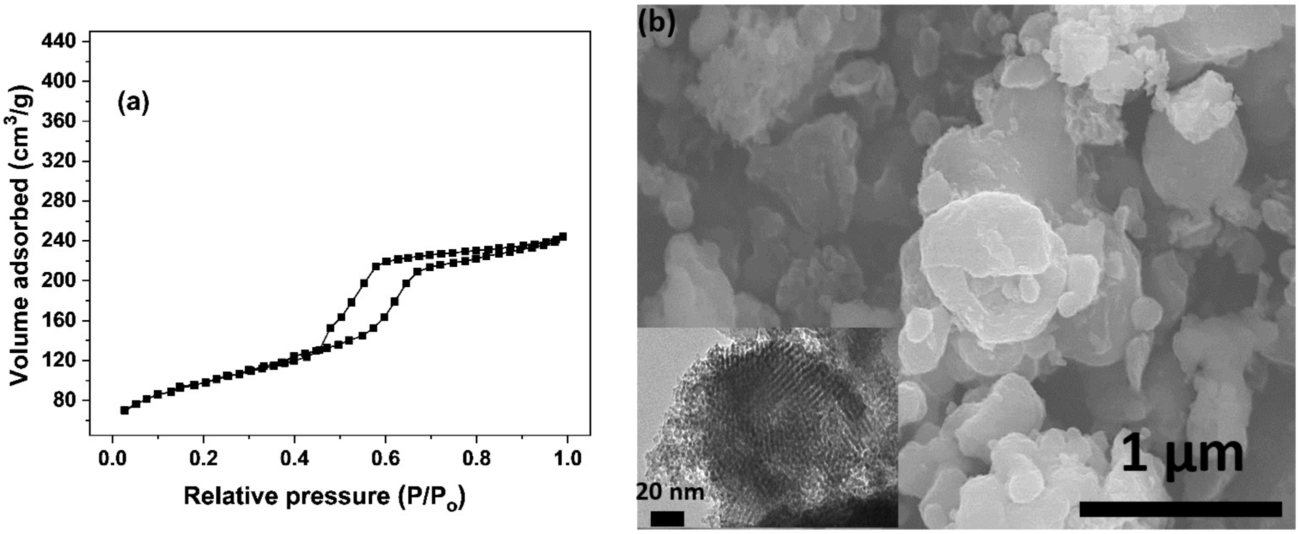

Figure S2. (**a**) N_2 sorption analysis; and (**b**) SEM and TEM (inset) images of control MSNs sample.

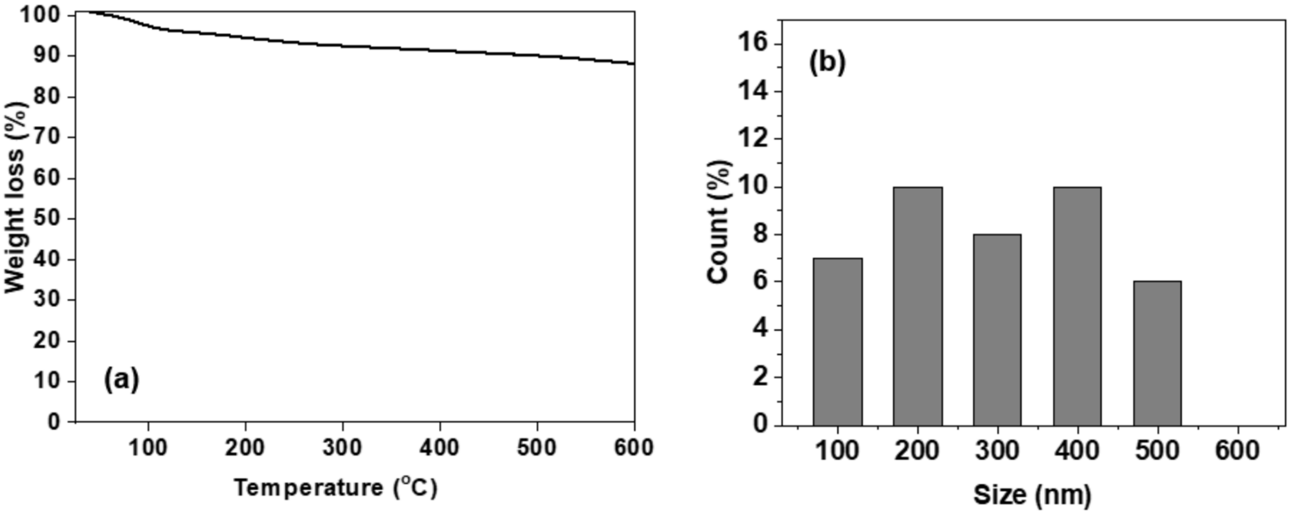

Figure S3. (**a**) TGA; and (**b**) DLS analysis of control MSNs sample.
